# Microbial Community Functional Change during Vertebrate Carrion Decomposition

**DOI:** 10.1371/journal.pone.0079035

**Published:** 2013-11-12

**Authors:** Jennifer L. Pechal, Tawni L. Crippen, Aaron M. Tarone, Andrew J. Lewis, Jeffery K. Tomberlin, M. Eric Benbow

**Affiliations:** 1 Department of Biology, University of Dayton, Dayton, Ohio, United States of America; 2 Southern Plains Agricultural Research Center, United States Department of Agriculture-Agricultural Research Service, College Station, Texas, United States of America; 3 Department of Entomology, Texas A & M University, College Station, Texas, United States of America; Oak Ridge National Lab, United States of America

## Abstract

Microorganisms play a critical role in the decomposition of organic matter, which contributes to energy and nutrient transformation in every ecosystem. Yet, little is known about the functional activity of epinecrotic microbial communities associated with carrion. The objective of this study was to provide a description of the carrion associated microbial community functional activity using differential carbon source use throughout decomposition over seasons, between years and when microbial communities were isolated from eukaryotic colonizers (e.g., necrophagous insects). Additionally, microbial communities were identified at the phyletic level using high throughput sequencing during a single study. We hypothesized that carrion microbial community functional profiles would change over the duration of decomposition, and that this change would depend on season, year and presence of necrophagous insect colonization. Biolog EcoPlates™ were used to measure the variation in epinecrotic microbial community function by the differential use of 29 carbon sources throughout vertebrate carrion decomposition. Pyrosequencing was used to describe the bacterial community composition in one experiment to identify key phyla associated with community functional changes. Overall, microbial functional activity increased throughout decomposition in spring, summer and winter while it decreased in autumn. Additionally, microbial functional activity was higher in 2011 when necrophagous arthropod colonizer effects were tested. There were inconsistent trends in the microbial function of communities isolated from remains colonized by necrophagous insects between 2010 and 2011, suggesting a greater need for a mechanistic understanding of the process. These data indicate that functional analyses can be implemented in carrion studies and will be important in understanding the influence of microbial communities on an essential ecosystem process, carrion decomposition.

## Introduction

Decomposition is an important component of nutrient and organic matter cycling in ecosystems [1]–[3] and forms the base of many food webs [4]. Microbial communities are ubiquitous in the biosphere [5]–[7]. Identifying the functional activity of microbial communities involved in the decomposition process can provide a mechanistic understanding into how detrital community ecology influences ecosystem processes [8]–[10]. Microbes are an important, but often understudied, trophic level in decomposition ecology. For instance, microbes contribute to the decay and recycling of plant organic matter and nutrients [11], which accounts for approximately 99% of the organic matter on Earth [12]. Microbes are also a major component of large carrion resource pulses such as salmon runs [13], [14] and cicada emergences [15], which are important in mediating ecosystem structure and function [16], [17]. However, often in such studies, decay rates or nutrient fluxes provide a measure of the overall decomposition process [10], with little attention given to the discrete function, specifically the carbon source use, of the associated microbial communities and their influence on decomposition rates [18]–[20].

Microbial communities associated with carrion are part of the necrobiome, *sensu* Benbow et al. [21]. These communities can be from the pre-existing living flora of the organism [22] and the environmental communities existing where carrion falls [23]. Those microbial communities associated with external surfaces of carrion are considered the epinecrotic microbial community, which has been hypothesized to regulate ecological interactions occurring during carrion decomposition through several mechanisms [24].

Prokaryotes can compete with eukaryotes by producing toxic compounds that affect resource “appeal” to consumers and thus reduce competition from eukaryotes [25]. Undisturbed microbial proliferation can deter consumption from higher trophic arthropods such as crustaceans [19] and insects [26]. Additionally, carrion microbial communities influence the physiology and behavior of necrophagous insects through the production of metabolically derived volatile molecules [9], [27]–[29]. Recent studies have documented potential interkingdom interactions with insects eavesdropping on microbial community communication; specifically, in laboratory studies insects have detected and behaviorally responded to quorum sensing and swarming associated molecules produced by bacteria [30], [31]. Yet, there is little understanding of how these interactions may occur in the natural environment, and how they may change during carrion decomposition. If metabolic by-products affect the arrival sequence of necrophagous insects (often the primary consumers of carrion) then microbial functional succession may define consumer community assembly and therefore mediate decomposition and the pathways of nutrient and energy recycling.

There are several factors influencing microbial community assembly and function during vertebrate carrion decomposition: temperature [32]–[34], moisture and humidity [35], tissue type [20], surrounding vegetation [36], [37] and soil pH [38]. However, there are a variety of challenges for researchers studying microbial assemblages during decomposition. Metabolic or physiological profiling is a practical and affordable approach to understand the functional role of microbes within the environment. The study of metabolomics uses a systematic approach to assess metabolic by-products for understanding biological processes [39]. The function of entire microbial communities can be assessed using techniques like Biolog EcoPlates™ [40], [41]. These phenotypic microarrays provide microbial metabolic community profiles (MMCPs) determined by the differential use of carbon sources from environmental samples [42], [43]. Plates similar to Biolog EcoPlates™ were originally developed for the medical field, and have had successful application in many ecological studies [42]–[46]. This technique has been used to study culturable microbial community functional activity in terrestrial [47]–[49] and aquatic habitats [45], [50], [51]. Biolog EcoPlates™ are ideal for evaluating the functional response of entire microbial communities, not individual species or taxa within the community, based on differential utilization of carbon sources (i.e., carbohydrates, polymers, carboxylic and acetic acids, amino acids, and amines/amides). Thus, this approach provides a community-level functional profile (i.e., MMCP) that can be compared between sample types or over time [51]. However, there are limitations to using this technique that have been previously highlighted (see review Preston-Mafham et al. 2011). Some of these limitations for heterogeneous environmental samples are related to the difficulty of quantifying and consistently maintaining the inoculation amount for each carbon source, the potential bias of dominant taxa outcompeting rare taxa present in the community and sensitivity to oxygen concentrations [52].

There are several emerging areas of research focused on a better understanding of microbial community functional ecology importance in carrion decomposition: 1) how does the functional activity of microbial communities change during decomposition; 2) the role of temperature driving this functional succession; 3) how microbial function is mediated by necrophagous insect colonization and succession; and 4) intra- and inter-seasonal variation in these processes [9], [27]. The overall aim of this study was to use Biolog EcoPlate™ MMCPs to characterize microbial community function throughout carrion decomposition, which is the first time the microbial metabolic activity will be characterized in carrion decomposition systems and assess that function with one data set of bacterial metagenomic sequence from matched samples. We tested three hypotheses: 1) microbial functional activity would be dependent on seasonal variation related to local ambient environmental (e.g., temperature) conditions; 2) microbial functional diversity would vary between years depending on temperatures; and 3) that necrophagous insect colonization would have a significant impact on microbial community functional change throughout carrion decomposition. The effects of necrophagous insects on microbial succession were further assessed during one field season by describing the structure of bacterial communities using 454-pyrosequencing with the objective to identify key phyla associated with community functional changes.

## Materials and Methods

### Seasonal Variation

Seasonal variation in MMCPs was studied within Morris Bean Reserve of Greene County, Ohio, USA (39° 45′53.67″ N, 83° 54′ 41.12″W), a Midwest temperate forest of 12.2 hectares surrounded by agricultural fields with a small tributary stream running adjacent to the reserve that empties into the Little Beavercreek River. The predominant trees were honey locust (*Gleditsia triacanthos*) and a variety of maples (*Acer* spp.), while the most common sub-canopy cover was Amur honeysuckle (*Lonicera maackii*). *Sus scrofa* (swine) carcasses weighing 14–18 kg were used as models of vertebrate carrion decomposition [53] during four seasonal trials: spring (March until June 2009), summer (July until August 2009), autumn (November 2009 until March 2010) and winter (February until March 2010).

Carcasses purchased from a local farm were double bagged during transport and prior to field placement to prevent insect colonization. Using the same experimental design of Benbow et al. [21], six replicate carcasses were used in the spring and summer trials whereas three were used for the autumn and winter trials; the latter trials only included three replicates because of cost constraints and longer decomposition time expected during the colder months. One carcass was placed randomly along five-1 m^2^ plots, along each of six transects (50 m) for a total of 30 plots within 10–80 m from each other as previously described [21], [54]. All carcasses were placed under anti-scavenging cages constructed of poultry netting wrapping a wooden frame (0.6×0.9×0.6 m) to prevent disturbance by large vertebrate scavengers (e.g., coyotes, vultures). Temperature was recorded every 0.25 h using NexSens DS1921G micro-T data loggers (Fondriest Environmental, Inc., Alpha, Ohio, USA). Temperature data were converted into accumulated degree hours (ADH), which accounts for temperature variation over decomposition time [55].

### Annual Variation and Necrophagous Insect Effects

Annual variation of microbial community activity and effects of necrophagous insect colonization was studied in a Midwestern temperate forest habitat surrounded by agricultural fields in Xenia, Ohio, USA (39° 38′14.83″ N, 83° 1′ 37.82″W). The dominant tree fauna consisted of oak (*Quercus* spp.) and maple. Carcasses ranging from 5–30 kg were sampled from 5 August 2010 until 14 August 2010 and 26 July 2011 until 2 August 2011. Carcasses purchased from the same farm as described above were double bagged during transport and prior to field placement to prevent insect colonization. In 2010, six male carcasses were randomly placed at a minimum of 20 m apart along three transects. In 2011, using the same methods as described in 2010, six carcasses (three females and three males) were purchased from the same farm, double bagged during transport to the field and randomly placed along three new transects.

During each year, three random carcasses were enclosed in individual 1.8 m^3^ Lumite® screen (18×14 mesh size) portable field cages (BioQuip Products, Rancho Dominguez, California, USA) to exclude insect colonization. These carcasses were considered the necrophagous insect exclusion treatment (EXC), while the three remaining carcasses were the insect access treatment (ACC) with insects immediately allowed access to the carcasses. As with the previous seasonal study, all carcasses were covered with anti-scavenging cages with NexSens DS1923 micro-T temperatures loggers measuring local ambient temperature every 0.25 h. Temperature data were converted into ADH.

Permission was granted by the Green County Park District for the seasonal trials at Morris Bean Reserve, while no permits were necessary for the annual and necrophagous insect colonization studies, as the landowners granted permission to use the privately owned property. The field studies did not involve endangered or protected species.

### Epinecrotic Microbial Community Sampling

During the seasonal trials, sterile cotton applicators were used to sample the microbial communities of the buccal cavity (the top area of the mouth and under the tongue), skin and the interior anal cavity. Epinecrotic microbial communities were sampled from the carcasses every three days, weather and field conditions permitting, until the dry stage of decomposition as defined by Payne [56].

In both the annual and necrophagous insect effect studies, sterile cotton applicators were used to sample microbial communities from two regions on each carcass for 60s: the buccal cavity and the skin, which consisted of combining three areas (approximately 2.54×15.24 cm) along a single transect of a carcass. Care was taken to assure that new areas were swabbed at subsequent samplings. Duplicate samples were collected from the carcasses for 16S rRNA analysis of the bacterial communities using 454-pyrosequencing during the first necrophagous insect effect study (2010). Carcasses were sampled until the dry stage of decomposition as described by Payne [56].

### Epinecrotic Microbial Function

Microbial community function was evaluated using phenotype Biolog EcoPlates™ (Biolog Inc., Hayward, California, USA) to provide MMCPs in all experiments [57], [58]. Biolog EcoPlates™ provide quantifiable functional responses of environmental microbial communities using 29 carbon sources (Table S1 in Materials S1); the tweens were used as a positive control to assess microbial growth on the plate and were excluded from analyses [58], [59]. The samples taken for seasonal microbial assessments were stored at −20°C for several months, slowly thawed and then processed using a modified protocol described by Insam and Goberna [60]. All samples from the annual and necrophagous insect effect studies were stored at 4°C and processed within 12 h. Briefly, samples were added individually to 50 ml Falcon tubes containing 40 ml of 25% Ringer solution and 15 sterilized 3 mm glass beads. All samples were homogenized using a Burrell Wrist-Action® shaker (Burrell Scientific, Pittsburg, Pennsylvania, USA) at the power ranking 9 for 10 min. Samples were centrifuged at 500×g for 2 min and the supernatant was retained. The plates were inoculated with 100 µl supernatant aliquots per well and incubated at 25°C in darkness based on preliminary experiments for these types of samples (unpublished data). Absorbance, or overall plate functional activity, was measured at 590 nm every 12 h up to 120 h or until the average plate absorbance reached 0.7 OD using a Wallac 1420 VICTOR^2^™ with Wallac 1420 Workstation software version 2.0 (Perkin Elmer, Inc., Waltham, Massachusetts, USA) during 2009 and 2010, and a Tecan Sunrise™ with Magellan™ software version 7.0 (Tecan Group Ltd., Männedorf, Switzerland) during 2011. A two-tailed paired t-test was used to test for any differences between the plate reader models, and there was not a significant difference in data between plate readers (t = 0.4620, df = 95, *P = *0.645).

### Data Analysis

After all seasonal samples were collected, three dates representing three different phases of decomposition (i.e., early, middle, and late) were chosen for analysis. The spring and summer trials followed the same sampling regime, with the first sampling date corresponding with the initial oviposition of Calliphoridae (Insecta: Diptera) signifying the early phase of decomposition (e.g., fresh and bloat). The second sampling date took place when the carcasses reached the middle phase of decomposition (e.g., active decay), while the last sampling date represented the late phase of decomposition (e.g., advanced decay and dry). For the autumn trial, the first sampling date corresponded with initial Calliphoridae oviposition; however, the second sampling date occurred prior to the first snow of the season. It was decided at the beginning of the study that carcasses would not be disturbed once they were covered with snow. The third sampling date took place in January of 2011, which corresponded with increased temperatures and snowmelt. For the winter trial, there were four sampling dates instead of three because of little insect activity during the beginning of the trial. The first sampling date took place immediately after carcass exposure since a delay in initial oviposition was expected during winter temperature conditions. The second date coincided with the next snowmelt. Both a third and fourth sampling date were chosen to represent different decomposition time points, even though there appeared to be negligible visible change to the carcasses and little adult or larval blow fly activity on the carcasses during the entire trial. Sampling for the annual and necrophagous insect colonizer effects studies occurred at initial field placement and on days 1, 3 and 5 during 2010. While in 2011, sampling occurred at initial field placement and then daily until 5 days of decomposition had occurred.

Analyses were performed according to Stefanowicz [44] and Weber and Legge [40]. To generate MMCPs, data were standardized by using the mean metabolic activity that approached or met 0.7 OD or after 10 consecutive readings. This was done to account for possible cell density differences among samples and provided a means to assess overall average microbial activity [40]. First, the average well color development, as determined by the absorbance measurement at 590 nm, was used to calculate the average carbon use of each plate. These data were then normalized using methods modified from Thottathil et al. [45] and Calbrix [46] by subtracting the average water well (background) activity for each plate, then subtracting that value from each carbon source usage, and then dividing by the average of all normalized carbon resources of the plate (Weber et al. 2010). The mean normalized metabolic activity for each carbon source was used for statistical analyses. A minimum base temperature of 0°C was assumed for these microbial community analyses as previously described [24].

### 454-Pyrosequencing of Communities

Samples collected in 2010 were used to identify microbial communities through 454-pyrosequencing using methods described in Pechal et al. [24]; a modified chloroform-phenol protocol was used for DNA extractions from each sample [24]. Samples were sent to Molecular Research Laboratories (Lubbock, TX) for bacterial tagged encoded FLX amplicon pyrosequencing. PCR amplification of V1–3 regions of 16S rRNA was performed using bacterial primers: Gray28F (5′ TTTGATCNTGGCTCAG) and Gray519r (5′GTNTTAC NGCGGCKGCTG) [61], [62]. The sequences have been deposited in the Sequence Read Archive at the European Bioinformatics Institute (accession number ERP001998). Taxonomic classification of the sequences were conducted using Naïve Bayesian rRNA classifier version 2.2 in the Ribosomal Database Project [63], [64]. The data from the ACC treatment in this study were presented in Pechal et al. [24], with a focus and analysis on family level modeling to estimate decomposition time. Here, we present phyletic data for both the ACC and EXC treatments as a qualitative comparison to functional profile changes over decomposition. For more specific (family) information on the ACC communities see Pechal et al. [24].

### Statistical Analyses

Bray-Curtis distance with nonmetric multidimensional scaling (NMDS) and 999 permutations using vegan 2.0–7 library in R [65] was used to visualize MMCP differences among seasons, sample dates, replicate carcasses, sample location (i.e., anal, buccal, skin) and treatment (EXC or ACC). Outliers were identified and removed prior to ordination using Jackknife distances in JMP 9.0.0 (SAS Institute Inc., Cary, NC, USA) as recommended by McCune and Grace [66]. For each of the ordinations, the two axes that explained the most variation with the strongest orthogonality (lowest stress) were used for representing the data in multidimensional space [66].

Differences in microbial function and variation over decomposition were tested using Bray-Curtis dissimilarity values with permutational multivariate analysis of variance (PERMANOVA) with the adonis function using the vegan 2.0–7 library in the R statistical package [65], [67]. PERMANOVA is a nonparametric technique used to differentiate groups of data based on a dissimilarity matrix [67].

## Results

### Seasonal Variation

**Figure 1:**
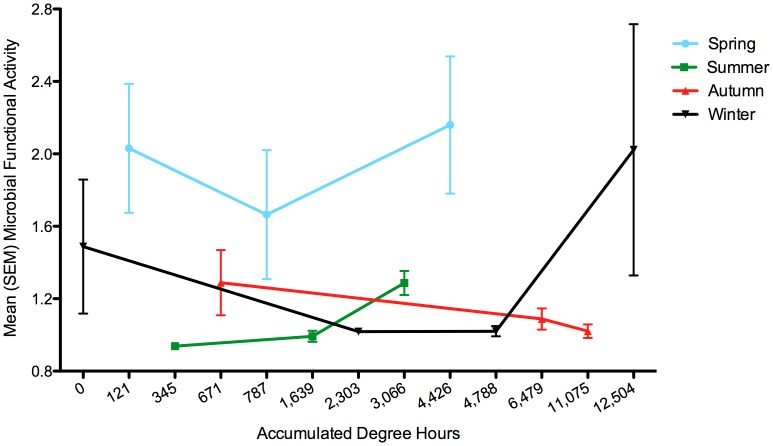
Seasonal microbial metabolic activity. The mean microbial functional activity for spring (blue circle), summer (green square), autumn (red triangle), and winter (black inverted triangle) over accumulated degree hours (ADH), which was used as a surrogate of decomposition time.

Spring overall microbial functional use of the carbon resources was highest of all seasons (Fig. 1) and increased by 6.0% from the first (121 ADH) to the last sampling day (4,426 ADH); the greatest functional activity change occurred during the summer with an increase of 27.1% from the first (345 ADH) to last (3,066 ADH) sampling day; autumn activity decreased by 20.9% over decomposition from 671 to 11,075 ADH; and winter microbial activity increased by 26.2% from 0 to 12,504 ADH.

Using PERMANOVA we found no significant differences in normalized MMCPs among the sample locations (buccal, skin, anus) of each carcass; therefore, data were pooled for the remainder of analyses. A two-dimensional NMDS ordination (stress = 0.198, R^2^ = 0.88) was sufficient to describe normalized carcass MMCPs among seasons (Fig. S1). There were significant differences (PERMANOVA: *P* = 0.001) in MMCPs among all seasons (Table 1; Fig. 1). Seasons were then separated to assess MMCP differences among the phases (early, middle and late) of decomposition (Fig. S2). There were significantly different MMCPs among decomposition phases in spring and summer (PERMANOVA: *P = *0.001), but no differences during the autumn (PERMANOVA: *P = *0.226) or winter studies (PERMANOVA: *P = *0.101).

**Table 1:** PERMANOVA results testing microbial community functional responses among seasons; and among carcass decomposition phases (i.e., early, middle and late), among seasons including the interaction with significant results indicated by an asterisk.

Factor	Source	d.f.	*SS*	MS	*F*	*P*
Season	Seasons	3	2.662	0.887	4.642	0.001*
	Residuals	162	30.970	0.191		
	Total	165	33.633			
Decomposition Phase	Seasons	3	2.662	0.887	5.595	0.001*
	DecompositionPhase	2	2.584	1.292	8.144	0.001*
	Season × DecompositionPhase	6	3.957	0.659	4.157	0.001*
	Residuals	154	24.430	0.159		
	Total	165	33.633			

### Annual Variation

There were significant differences in carcass MMCPs over decomposition, a significant difference between years, and no significant interaction (Table 2). Overall mean functional activity was significantly higher (PERMANOVA: *P*<0.001) in 2011 (Fig. S3). A two-dimensional NMDS ordination (stress = 0.199, R^2^ = 0.87) described the variation in carcass MMCPs between years (Fig. S4).

**Table 2:** PERMANOVA results testing microbial community functional responses between years and among carcass decomposition days including the interaction with significant results indicated by an asterisk.

Factor	Source	d.f.	*SS*	MS	*F*	*P*
Year	Year	1	1.6909	1.69088	9.1259	0.001*
	Decomposition Day	5	1.5231	0.30462	1.6441	0.007*
	Season × Decomposition Day	3	0.7220	0.24067	1.2989	0.113
	Residuals	93	17.2313	0.18528		
	Total	102	21.1673			

### Annual Variation and Necrophagous Insect Effects

#### 2010

**Figure 2:**
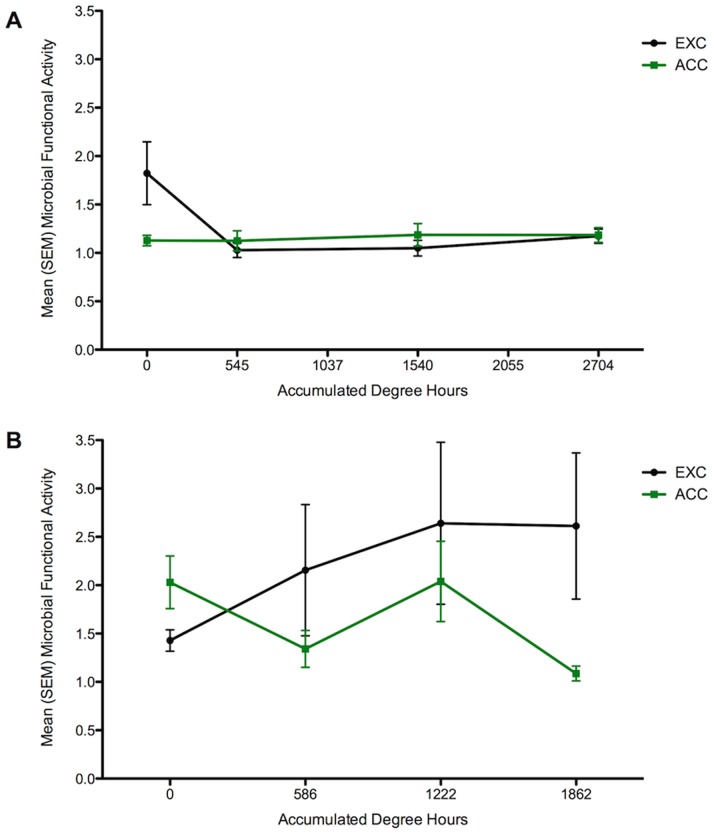
Epinecrotic microbial metabolic activity with delayed initial insect colonization. The mean microbial functional activity in the A) 2010 field trial for carcasses excluded from insect access (EXC-black circle) and carcasses allowed to have insect access (ACC-green square), and in B) 2011 field trial for carcasses excluded from insect access (EXC-black circle) and carcasses allowed to have insect access (ACC-green square) over accumulated degree hours (ADH), which was used as a surrogate of decomposition time.

There was a significant difference in carcass MMCPs over decomposition, no difference between treatments (EXC and ACC) and a significant interaction effect (Table 3). Overall, mean microbial functional activity of ACC carcasses increased by 4.2% over decomposition; however, it decreased by 35.7% in EXC carcasses (Fig. 2). There were significant differences in MMCPs over decomposition and between sampling regions (buccal and skin), with no significant interaction effects (Table S2 in Materials S1); buccal community microbial activity initially decreased on sampling day 1 followed by a slight increase in activity until the fifth sampling day with an overall net decrease from the initial to last sampling day (Fig. S5). Skin communities exhibited a similar pattern of decreased overall microbial activity until sampling day 3 followed by increased activity as decomposition progressed with an overall net decrease in microbial activity from the initial to last sampling day (Fig. S5).

**Table 3:** PERMANOVA results testing microbial community functional responses between treatments (EXC and ACC) and among carcass decomposition days including the interaction for both 2010 and 2011 field seasons with significant results indicated by an asterisk.

Factor	Source	d.f.	*SS*	MS	*F*	*P*
2010	Treatment	1	0.1417	0.14170	1.0998	0.353
	Decomposition Day	3	0.8350	0.27833	2.1603	0.001*
	Treatment × Decomposition Day	3	0.5906	0.19687	1.5280	0.017*
	Residuals	40	5.1537	0.12884		
	Total	47	6.7211			
2011	Treatment	1	0.0743	0.0743	0.3249	0.991
	Decomposition Day	5	1.4353	0.2871	1.2550	0.139
	Treatment × Decomposition Day	3	0.9527	0.3176	1.3884	0.104
	Residuals	45	10.2931	0.2285		
	Total	54	12.7554			

A two-dimensional NMDS ordination (stress = 0.177, R^2^ = 0.91) was sufficient to describe the variation in carcass MMCPs between ACC and EXC carcasses (Fig. S6). There were not significantly different MMCPs between insect exclusion and insect access carcasses, but there was a significant difference in MMCPs over decomposition time and a significant interaction (Table 3); thus data from each treatment were pooled for further analysis. Pair-wise comparisons indicated significantly different MMCPs between the initial day (Day 0) and each subsequent day of decomposition (Days 1, 3 and 5).

### 454-Pyrosequencing of Communities

**Figure 3:**
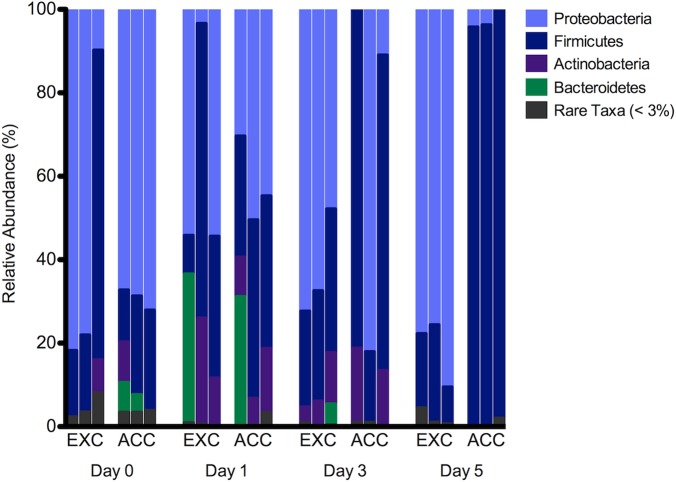
Identification of carcass microbial communities over decomposition using 454-pyrosequencing. Phyletic level taxonomic relative abundance of the microbial communities based on 454-pyrosequencing between treatments (EXC and ACC) over decomposition days during 2010. Day 0 is the initial field placement followed by subsequent sample collections on days 1, 3 and 5 post-field placement. Rare taxa includes any phyla with <3% of the total relative abundance.

A total of 378,904 sequences were obtained throughout carcass decomposition. At initial field placement, Proteobacteria was the predominant phyla for EXC (62%) and ACC (70%) carcasses with Firmicutes being the next predominant phyla at 33% and 20% of EXC and ACC communities, respectively. Proteobacteria remained predominant on the first (35%), third (63%), and fifth (82%) sampling days for EXC carcasses. While Firmicutes decreased as decomposition progressed on the first (43%), third (28%), and fifth (16%) day. However, ACC displayed an inverse trend as Proteobacteria decreased from the first (42%) and third (44%) days to only 3% total abundance on the fifth day, but with some inter-carcass variation as discussed in detail in Pechal et al. [24]. Firmicutes was the predominant phyla as decomposition progressed comprising of 36, 48, and 96% on the first, third and fifth sampling day, respectively. Rare phyla accounted for less than 0.5% of the total relative abundance across decomposition (Fig. 3).

#### 2011

Over decomposition, mean microbial functional activity of ACC carcasses decreased by 46.3% while there was a 45.2% increase on EXC carcasses (Fig. 2). There were no significant differences in carcass MMCPs over decomposition, between treatments nor was there a significant interaction (Table 3). However, there was a significant difference in microbial functional activity between the buccal and skin communities and the composite sample but there was no significant difference over decomposition and no significant interaction. The buccal communities increased on day 1, decreased by day 3, and increased on day 5 with an overall net increase in mean microbial activity from the initial to last sampling day (Fig. S5). Skin communities exhibited an inverse trend of decreased activity on day 1, increased by day 3, and finally decreased on day 5 with an overall net decrease in mean microbial activity from the initial to last sampling day (Fig. S5). However, there were no significant differences in MMCPs between sampling regions (buccal, skin), but a significant interaction (Table S2 in Materials S1).

A two-dimensional NMDS ordination (stress = 0.166, R^2^ = 0. 89) was sufficient to describe the variation in carcass MMCPs between ACC and EXC carcasses (Fig. S6).

### Abiotic Conditions

Mean daily ambient temperature among carcasses was 23.2±2.1°C during 2010 and 25.1±1.0°C in 2011. Mean ADH associated with the carcasses in 2011 was significantly higher (F_1,7_ = 432.9, *P*<0.001) by 8–18% than 2010 (data not shown) throughout decomposition, except at initial placement in the field when ADH for all carcasses was zero.

## Discussion

Here we report the use of Biolog EcoPlates™ for describing microbial community metabolic profiles throughout carrion decomposition as a representation of the overall functional activity and differentiated carbon source use of epinecrotic communities. The results demonstrated 1) seasonal variation of microbial community function, with overall higher and more variable activity during spring when compared to the other seasons; 2) annual differences in metabolic profiles; and 3) inconsistent effects of necrophagous insect activity (e.g., blow fly oviposition and subsequent larval development) on microbial functional profiles and overall activity.

Fungi and bacteria have been documented as facilitators of the initial decomposition processes of carrion [19], [68]. The inherent stochastic spatial and temporal nature of microbial communities on ephemeral resources, such as carrion, may contribute to the variation both within and across carcasses [69]. We demonstrated that microbial metabolic community profiles varied by season, and within a season they were highly variable during the carcass decomposition process. We also found that when initial insect access and colonization was delayed the metabolic profile changes that occurred during decomposition depended on the year of study, while overall metabolic activity was more highly variable for microbial communities of insect exclusion carcasses during both years. These results suggest that microbial abundance and activity of epinecrotic communities are patchy and sensitive to environmental factors such as temperature and habitat. This variability in metabolic profile responses both within and between seasons and years may be tied to corresponding shifts in the microbial community composition throughout the decomposition process, but this requires additional study. There are limitations associated with sole-carbon source utilization profiles [52]; however, in this study metabolic profiles were used as a potential surrogate for differentiating microbial community changes on the resource over time (e.g., decomposition).

### Seasonal Variation

Seasonal variance in abiotic conditions was hypothesized to significantly impact microbial community function. For the spring and winter trials, microbial functional activity decreased during early decomposition followed by an increase in later phases of decomposition. The increase in functional activity during the winter trial most likely results from a lack of competition with invertebrates (e.g., blow fly larvae) because of the colder temperatures (i.e., <5°C). Summer had an increase in microbial activity throughout decomposition. Autumn was the only season that demonstrated a decreasing trend in overall activity throughout decomposition, which can be due to competition with invertebrates, the depletion of the carrion resource, or both. Competition between microbes and insects for a resource occurs by microbes producing toxins that deter eukaryotic consumers [25], while insects can produce antimicrobial peptides that inhibit microbial proliferation [70].

We found significantly different metabolic profiles over decomposition for carcasses in spring and summer but not autumn and winter. However, why did microbial functional activity increase in the winter trial as temperatures decreased? Carter and Tibbett [33] demonstrated when chicken carcasses were subjected to different temperatures, the efficiency of the microbial metabolic activity (respiration) during decomposition varied. They reported that when carcasses decomposed under 2, 12, and 22°C, the process was most efficient at 2°C. This may explain the increased functional activity between the second and last sampling dates for the winter trial, but this requires additional investigation into the optimal metabolic temperatures of multi-species assemblages of microbes. This could also aide in explaining microbial functional variability in different seasons. For the spring and summer trials, large Calliphoridae larval masses colonized and consumed the carcass throughout the decomposition process. Carter and Tibbett [33] also speculated that higher ambient temperatures might have negative impacts on microbial community catabolic reactions. Dipteran larval masses have the potential to generate increased temperatures up to 50°C higher than the surrounding ambient temperature through collective thermogenesis [71]. The depletion of the carrion resource by an invertebrate competitor, but also increased aggregate temperatures resulting from calliphorid larval masses may be affecting microbial community assembly and overall activity during carrion decomposition. Additional studies are warranted to further understand this process.

### Annual Variation

Qualitative phases of decomposition could be differentiated for carcasses based on microbial functional activity, and insects appeared to be reducing the microbial community throughout the decomposition process in 2010. However, these trends were not consistent between years. The lack of similarities between years may result from abiotic (e.g., temperature) and biotic factors (e.g., pre-existing microbial communities), as was demonstrated in our seasonal study. Abiotic factors including temperature may account for microbial community function variation between years and throughout decomposition. Ambient temperatures were up to 18% higher in 2011. Previous studies, however, have reported conflicting microbial species response to changing temperature. For instance, soil bacteria abundance increased with temperature (+3°C) in the presence of elevated carbon dioxide, but decreased under conditions of similar temperature conditions without elevated carbon dioxide [72]. Therefore, during 2011, higher average temperature may have been associated with increased available carbon and overall activity of the carcass microbial communities regardless of treatment. Finally, the variation may be associated with other parameters or interactions (e.g., pH changes occurring as part of the carrion or soil) that were not assessed during this study [73].

### Necrophagous Insect Effects

Primary insect colonizers utilize the carrion resource as nutrition, mating or an oviposition site. Subsequent larval development may disrupt established microbial communities through direct or indirect competitive interactions on the carcass [25], [70]. Blow flies may directly impact microbial species through chemical secretions while consuming carrion tissue as changes in the microbial community have been demonstrated when using calliphorid larvae for wound debridement therapy [74], [75]. Insects arriving to colonize carrion could introduce their own exogenous microbial community [76], such as *Musca domestica* (Diptera: Muscidae), which carries over 100 pathogenic microbes [77], [78]. The introduction of insect associated microbial communities may influence carrion microbial community function through microbially mediated competitive mechanisms, which can alter metacommunity dynamics and biogeographic patterns of microbial communities in the landscape [79], [80]. For example, one of the Biolog EcoPlates™ carbon sources, putrescine, is a well know volatile associated with decomposing remains [81]–[84] and is a blow fly attractant but repellant for male carrion beetles (Coleoptera: Silphidae) [85]. Putrescine is also a molecule required for the swarming behavior of bacteria such as *Proteus mirabilis*, which is commonly associated with blow flies [30], [31]. Alternatively, specific microbes or functional groups may be outcompeting other species present during later phases of undisturbed decomposition, which has been found in other systems [86], [87]. In this study, we found that overall functional activity was affected by the initial access or exclusion of insect colonizers, but the response depended on the year of study, and presumably associated temperature conditions. These functional responses were also associated with the phyletic differences in communities associated with insect exclusion, suggesting 1) there is variability in the structure and function of carrion microbial communities and 2) that necrophagous flies have important effects on microbial community assembly during decomposition. A similar pattern has been observed for undisturbed vernal rain pools that produced significantly higher species richness of protozoan and metazoan species [88] and higher protozoan and rotifer richness in undisturbed artificial container communities filled by rain than that found in disturbed vernal pools [89], [90]. While the variation of microbial metabolic profiles among years and in the presence of necrophagous insects is not surprising, these insect-microbe interactions are still poorly understood in carrion decomposition systems.

Additionally, we documented four major phyla associated with the carcasses throughout decomposition using 454-pyrosequencing that included Proteobacteria, Firmicutes, Actinobacteria and Bacteroidetes. These phyla have been reported in one other study where epinecrotic communities were described using 454-pyrosequencing (Pechal et al. [24]). Taxa within Proteobacteria are commonly associated with the spoiling of meat and are found on the hides of slaughtered animals [91]. While taxa in the remaining three phyla are associated with the human microbiome [92] and soil communities [93]. Thus, shifts in functional profiles exhibited throughout the 2010 field trial may have resulted from the changes in the relative abundance of these four main phyla. It is unknown at this time whether or not there is a difference of species composition or functional groups based on taxonomic identification of bacteria.

### Conclusions

These results demonstrate for the first time the use of metabolic profiling to assess carrion decomposition, and that there is great potential to use this technique for carrion decomposition research. Current research in this field has been aimed at determining what microorganisms compose communities involved in carrion decomposition [19], [20]. Our results demonstrate that the microbial metabolic profiles describe significant functional changes in the community during decomposition both within and among seasons, similar to other studies in aquatic habitats [19], [20]. This demonstrates community-level functional changes occurring over decomposition; however, more detailed research involving which species are changing over time is needed using high throughput metagenomic sequencing approaches [94], [95]. Specifically, using microbial functional data in conjunction with metagenomic and entomological data is needed to better understand the complexity and importance of the necrobiome to decomposition.

Overall, empirical data are sparse within the microbe-insect-carrion model in terrestrial ecosystems. We have demonstrated that insects may have moderating effects on decomposition by mediating microbial structure and function. It is important to further investigate the role of microbes and their importance in determining underlying mechanisms controlling community assembly, biomass turnover and nutrient cycling of ephemeral resources.

## Supporting Information

Figure S1 **Seasonal** **Bray-Curtis dissimilarity based non-metric multidimensional scaling.** Using Bray-Curtis dissimilarity values, MMCPs for seasonal carrion communities were plotted using a two dimensional non-metric multidimensional scaling model (stress = 0.198, R^2^ = 0.88). The MMCPs were significantly different (PERMANOVA: *P = *0.001) among seasons. The circles indicate 95% standard error of each season.
(TIFF)

Figure S2 **Decomposition phase within season Bray-Curtis dissimilarity based non-metric multidimensional scaling.** Non-metric multidimensional scaling ordinations of MMCPs were plotted on two dimensions for carrion communities of decomposition phases (early, middle and late) during A) spring (stress = 0.122, R^2^ = 0.94), B) summer (stress = 0.098, R^2^ = 0. 98), C) autumn (stress = 0.149, R^2^ = 0.91), and D) winter (stress = 0.158, R^2^ = 0.91) seasons. There were significant differences among decomposition phases in spring and summer (PERMANOVA: *P = *0.001), but no significant difference among decomposition phases in autumn (PERMANOVA: *P = *0.226) or winter (PERMANOVA: *P = *0.011). The circles indicate 95% standard error of each decomposition phases.
(TIFF)

Figure S3 **Mean functional activity over decomposition between years.** The mean (SEM) microbial functional activity between the 2010 (gray circle) and 2011 (black square) field trials at initial field placement (Day 0) and subsequent sampling on days 1, 3, and 5.
(TIFF)

Figure S4 **Annual Bray-Curtis dissimilarity based non-metric multidimensional scaling.** Using Bray-Curtis dissimilarity values, MMCPs from both 2010 and 2011 field seasons were plotted using a two dimensional non-metric multidimensional scaling model (stress = 0.199, R^2^ = 0.87). There were significantly different MMCPs (PERMANOVA: *P = *0.001) between years. The circles indicate 95% standard error of each year.
(TIFF)

Figure S5 **Mean functional activity over decomposition between sampling regions in both field seasons.** The mean microbial functional activity for the buccal (orange circle) and skin (yellow square) sampling region in the A) 2010 and B) 2011 field trials at initial field placement (Day 0) and subsequent sampling on days 1, 3, and 5.
(TIFF)

Figure S6 **Treatment within field season Bray-Curtis dissimilarity based non-metric multidimensional scaling.** Non-metric multidimensional scaling ordinations of MMCPs were plotted on two dimensions for ACC and EXC carrion communities during A) 2010 (stress = 0.177, R^2^ = 0.91), and B) 2011 (stress = 0.166, R^2^ = 0. 86) field seasons.
(TIFF)

Materials S1 **Supplementary Tables S1–S2. Table S1,** Carbon sources and their respective groupings (i.e., amines/amides, amino acid, carbohydrate, and carboxylic and acetic acid) in a Biolog EcoPlate™. **Table S2,** PERMANOVA results testing MMCPs between sampling region (buccal and skin) communities and among carcass decomposition days including the interaction for both 2010 and 2011 field seasons with significant results indicated by an asterisk.
(DOCX)
