# Supplementary figures and images for: Microbial Community Functional Change during Vertebrate Carrion Decomposition
Source: PLoS One. 2013 Nov 12;8(11):e79035. doi: 10.1371/journal.pone.0079035 (PMC3827085; doi:10.1371/journal.pone.0079035)

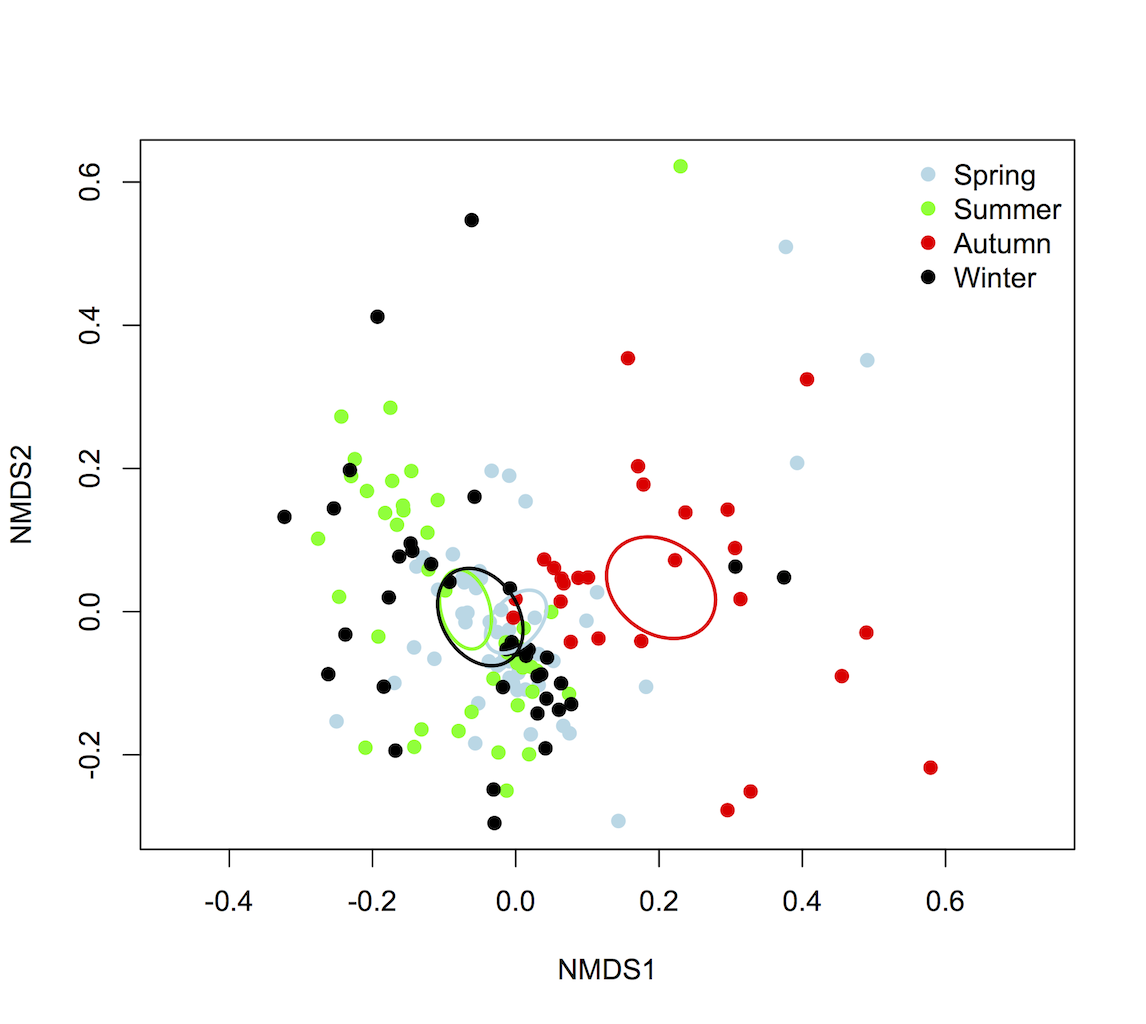

Supplement: Figure S1 — Seasonal Bray-Curtis dissimilarity based non-metric multidimensional scaling. Using Bray-Curtis dissimilarity values, MMCPs for seasonal carrion communities were plotted using a two dimensional non-metric multidimensional scaling model (stress = 0.198, R2 = 0.88). The MMCPs were significantly different (PERMANOVA: P = 0.001) among seasons. The circles indicate 95% standard error of each season. (TIFF) [file pone.0079035.s001.tiff]

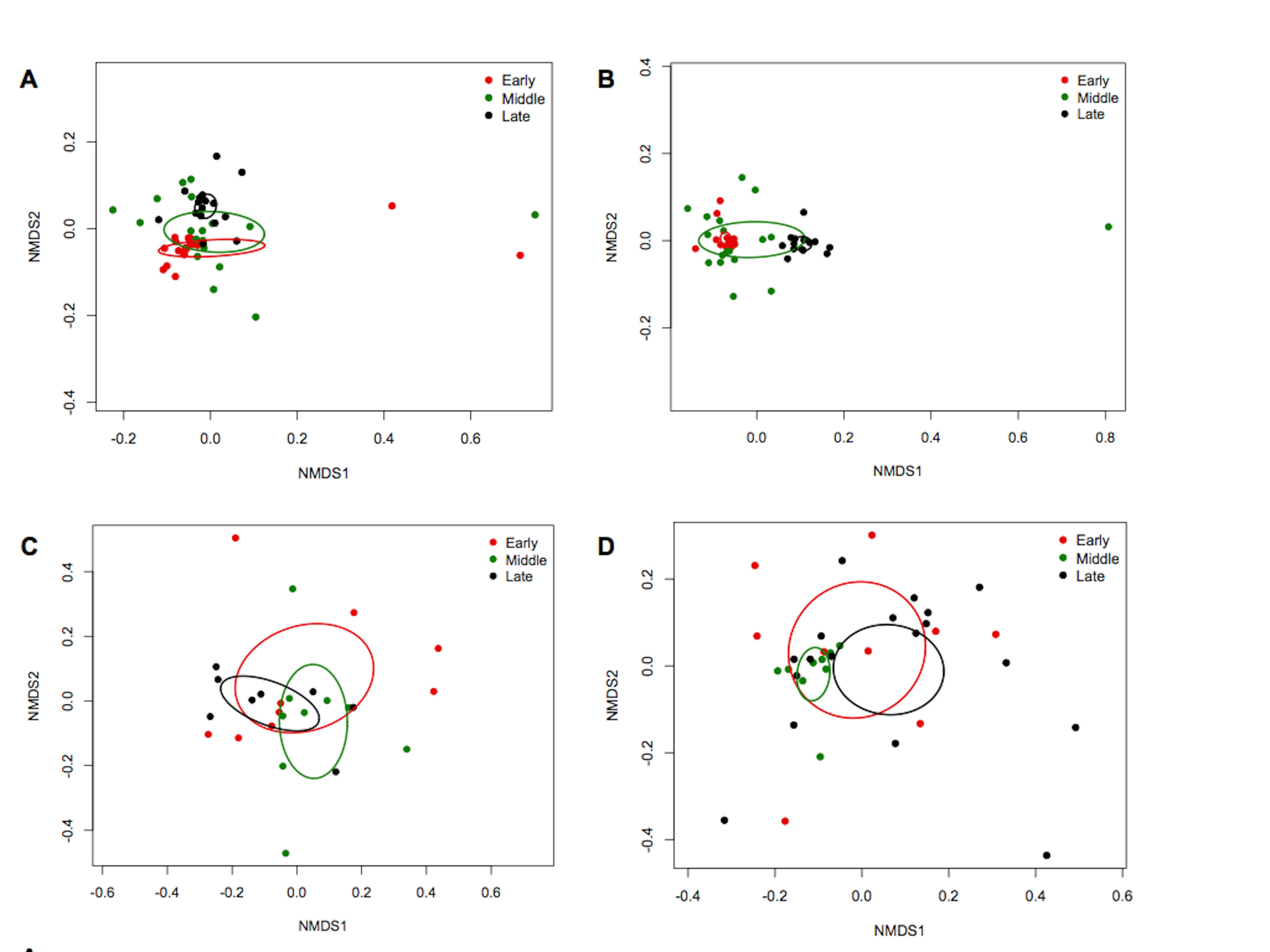

Supplement: Figure S2 — Decomposition phase within season Bray-Curtis dissimilarity based non-metric multidimensional scaling. Non-metric multidimensional scaling ordinations of MMCPs were plotted on two dimensions for carrion communities of decomposition phases (early, middle and late) during A) spring (stress = 0.122, R2 = 0.94), B) summer (stress = 0.098, R2 = 0. 98), C) autumn (stress = 0.149, R2 = 0.91), and D) winter (stress = 0.158, R2 = 0.91) seasons. There were significant differences among decomposition phases in spring and summer (PERMANOVA: P = 0.001), but no significant difference among decomposition phases in autumn (PERMANOVA: P = 0.226) or winter (PERMANOVA: P = 0.011). The circles indicate 95% standard error of each decomposition phases. (TIFF) [file pone.0079035.s002.tiff]

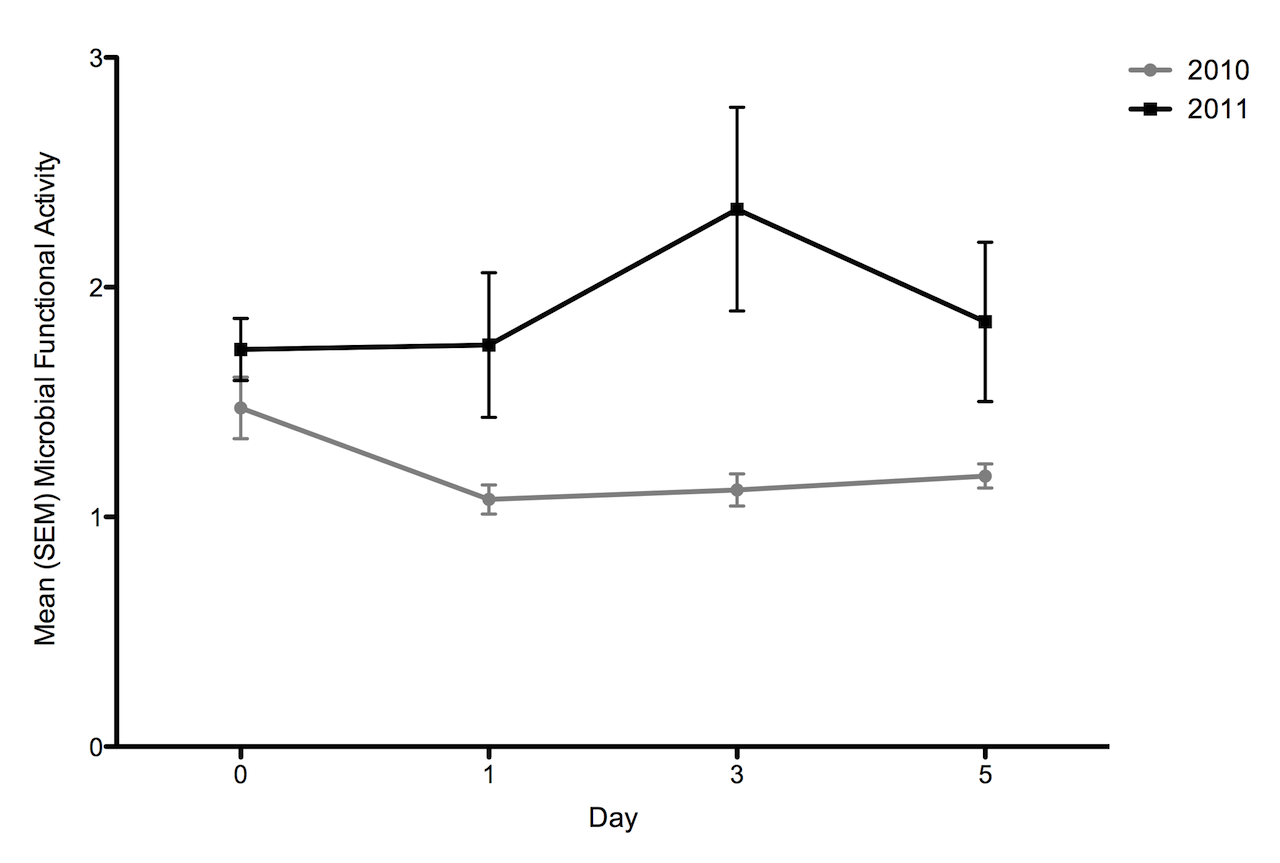

Supplement: Figure S3 — Mean functional activity over decomposition between years. The mean (SEM) microbial functional activity between the 2010 (gray circle) and 2011 (black square) field trials at initial field placement (Day 0) and subsequent sampling on days 1, 3, and 5. (TIFF) [file pone.0079035.s003.tiff]

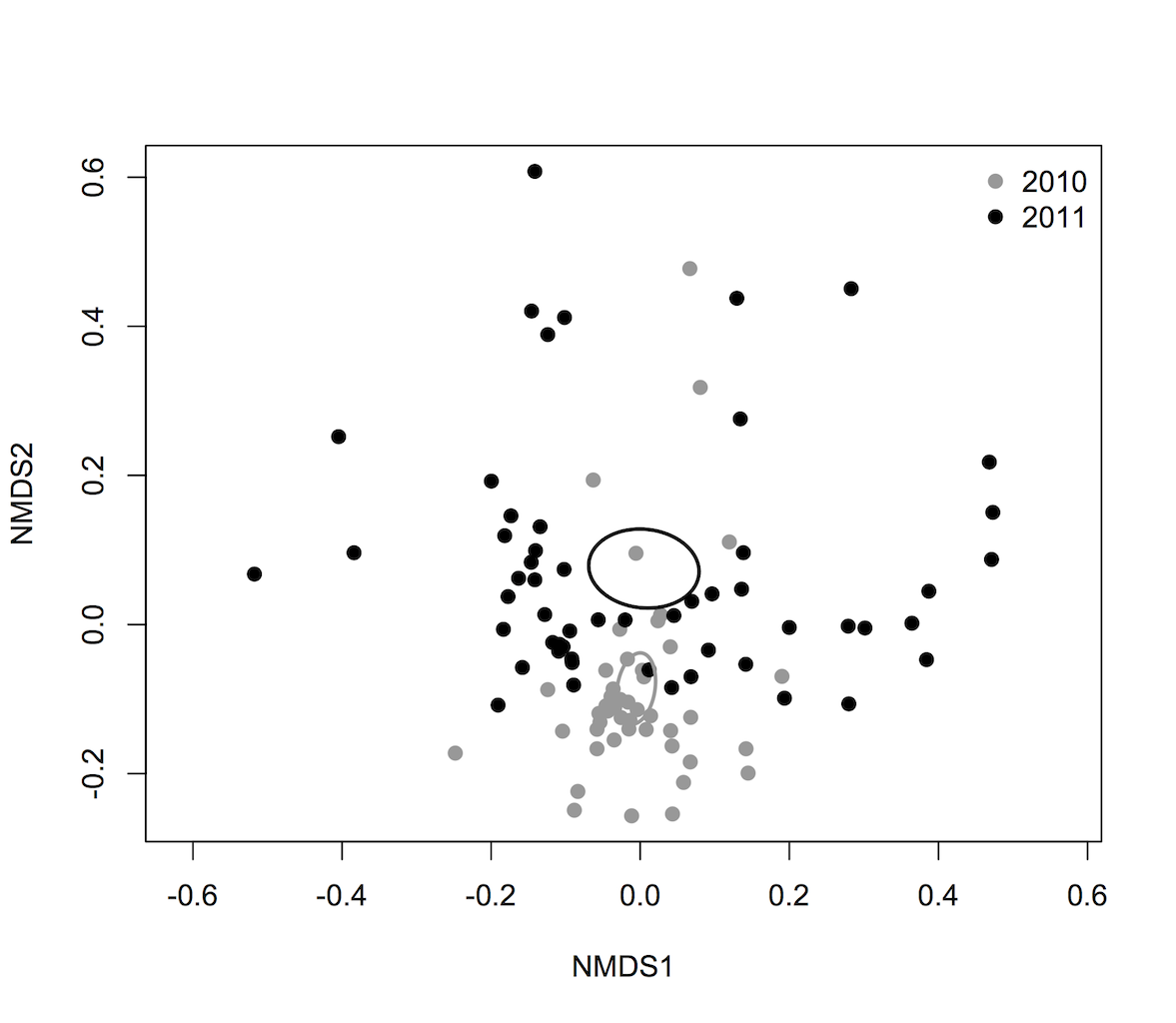

Supplement: Figure S4 — Annual Bray-Curtis dissimilarity based non-metric multidimensional scaling. Using Bray-Curtis dissimilarity values, MMCPs from both 2010 and 2011 field seasons were plotted using a two dimensional non-metric multidimensional scaling model (stress = 0.199, R2 = 0.87). There were significantly different MMCPs (PERMANOVA: P = 0.001) between years. The circles indicate 95% standard error of each year. (TIFF) [file pone.0079035.s004.tiff]

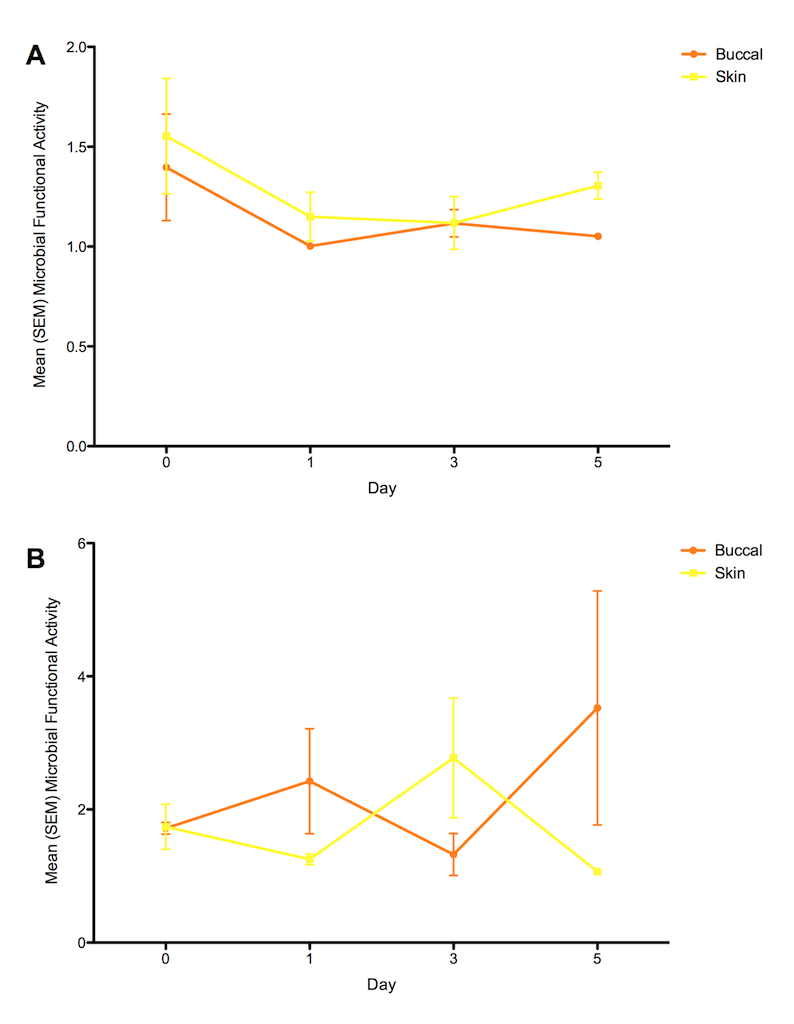

Supplement: Figure S5 — Mean functional activity over decomposition between sampling regions in both field seasons. The mean microbial functional activity for the buccal (orange circle) and skin (yellow square) sampling region in the A) 2010 and B) 2011 field trials at initial field placement (Day 0) and subsequent sampling on days 1, 3, and 5. (TIFF) [file pone.0079035.s005.tiff]

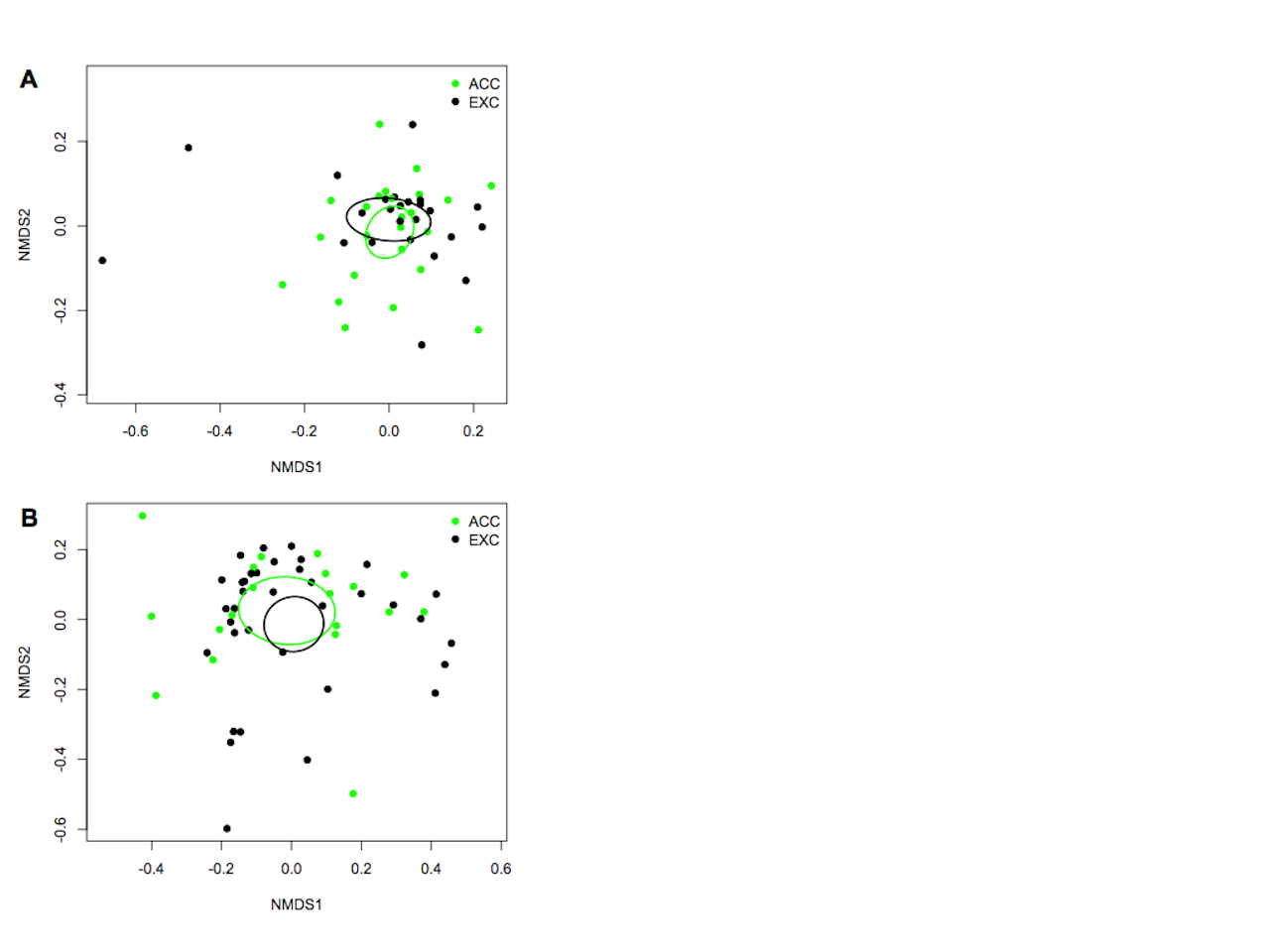

Supplement: Figure S6 — Treatment within field season Bray-Curtis dissimilarity based non-metric multidimensional scaling. Non-metric multidimensional scaling ordinations of MMCPs were plotted on two dimensions for ACC and EXC carrion communities during A) 2010 (stress = 0.177, R2 = 0.91), and B) 2011 (stress = 0.166, R2 = 0. 86) field seasons. (TIFF) [file pone.0079035.s006.tiff]
