# Supplementary material for: Microbial Community Functional Change during Vertebrate Carrion Decomposition
Source: PLoS One. 2013 Nov 12;8(11):e79035. doi: 10.1371/journal.pone.0079035 (PMC3827085; doi:10.1371/journal.pone.0079035)
Supplement: Materials S1 — Supplementary Tables S1–S2. Table S1, Carbon sources and their respective groupings (i.e., amines/amides, amino acid, carbohydrate, and carboxylic and acetic acid) in a Biolog EcoPlate™. Table S2, PERMANOVA results testing MMCPs between sampling region (buccal and skin) communities and among carcass decomposition days including the interaction for both 2010 and 2011 field seasons with significant results indicated by an asterisk. (DOCX) [file pone.0079035.s007.docx]

**Table S1** Carbon sources and their respective groupings (i.e., amines/amides, amino acid, carbohydrate, and carboxylic and acetic acid) in a Biolog EcoPlate™ [[1](#_ENREF_1),[2](#_ENREF_2)].

| **Carbon** | **Grouping** |
| --- | --- |
| Phenylethylamine | Amines/amides |
| Putrescine | Amines/amides |
| Glycyl-L-Glutamic Acid | Amino acid |
| L-Arginine | Amino acid |
| L-Asparagine | Amino acid |
| L-Phenylalanine | Amino acid |
| L-Serine | Amino acid |
| L-Threonine | Amino acid |
| D-Cellobiose | Carbohydrate |
| D-Mannitol | Carbohydrate |
| D-Xylose | Carbohydrate |
| DL-α-Glycerol Phosphate | Carbohydrate |
| Glucose-1-Phosphate | Carbohydrate |
| i-Erythritol | Carbohydrate |
| N-Acetyl-D-Glucosamine | Carbohydrate |
| Pyruvic Acid Methyl Ester | Carbohydrate |
| α-D-Lactose | Carbohydrate |
| β-Methyl-D-Glucoside | Carbohydrate |
| 2-Hydroxy Benzoic Acid | Carboxylic and Acetic acid |
| 4-Hydroxy Benzoic Acid | Carboxylic and Acetic acid |

**Table S1** Continued.

| **Carbon** | **Grouping** |
| --- | --- |
| D-Galactonic Acid λ-Lactone | Carboxylic and Acetic acid |
| D-Galacturonic Acid | Carboxylic and Acetic acid |
| D-Glucosaminic Acid | Carboxylic and Acetic acid |
| D-Malic Acid | Carboxylic and Acetic acid |
| Itaconic Acid | Carboxylic and Acetic acid |
| α-Ketobutyric Acid | Carboxylic and Acetic acid |
| λ-Hydroxybutyric Acid | Carboxylic and Acetic acid |
| Glycogen | Polymer |
| α-Cyclodextrin | Polymer |

**Table S2** PERMANOVA results testing MMCPs between sampling region (buccal and skin) communities and among carcass decomposition days including the interaction for both 2010 and 2011 field seasons with significant results indicated by an asterisk.

| **Factor** | **Source** | **d.f.** | ***SS*** | **MS** | ***F*** | ***P*** |
| --- | --- | --- | --- | --- | --- | --- |
| 2010 | Region | 1 | 0.3347 | 0.33475 | 2.6210 | 0.004* |
|  | Decomposition Day | 3 | 0.8350 | 0.27833 | 2.1793 | 0.001* |
|  | Region x Decomposition Day | 3 | 0.4426 | 0.14753 | 1.1551 | 0.217 |
|  | Residuals | 40 | 5.1087 | 0.12772 |  |  |
|  | Total | 47 | 6.7211 |  |  |  |
|  |  |  |  |  |  |  |
| 2011 | Region | 1 | 0.2828 | 0.28278 | 1.2999 | 0.195 |
|  | Decomposition Day | 5 | 1.3937 | 0.27873 | 1.2813 | 0.133 |
|  | Region x Decomposition Day | 5 | 1.7245 | 0.34491 | 1.5855 | 0.021* |
|  | Residuals | 45 | 9.3544 | 0.21754 |  |  |
|  | Total | 54 | 12.7554 |  |  |  |

References

1. Weber KP, Legge RL (2009) One-dimensional metric for tracking bacterial community divergence using sole carbon source utilization patterns. Journal of Microbiological Methods 79: 55-61.

2. Zak JC, Willig MR, Moorhead DL, Wildman HG (1994) Functional diversity of microbial communities: A quantitative approach. Soil Biology and Biochemistry 26: 1101-1108.
